# Supplementary material for: In Silico Reconstruction of Sperm Chemotaxis
Source: Int J Mol Sci. 2021 Aug 24;22(17):9104. doi: 10.3390/ijms22179104 (PMC8431315; doi:10.3390/ijms22179104)
Supplement: Supplementary file 1 [file ijms-22-09104-s001.zip › ijms-1335052-supplementary.pdf]

Supporting Information 1

Source code of the chemotaxis simulation with sea urchin model

```
#include <stdio.h>
#include <math.h>
#include <stdlib.h>
#include <time.h>
#include "MT.h"

/*This function detects the arrival of sperm to the egg*/
double rch(float a, float b)
{
    if(sqrt(a*a+b*b) < 100)
        {
            return 1;
        }
    else
        {
            return 0;
        }
}

/*This function generates random number with normal distribution*/
double rand_normal(double mu, double sigma)
{
    double z;
    double a;
    double b;
    a=genrand_real3();
    b=genrand_real3();
    z=sqrt(-2*log(a))*sin(2*M_PI*b);
    return mu+sigma*z;
}

/*This is a main function for simulation*/
int main(void)
{
```

```
/*declaration of variables*/
int t=0; /*unit time*/
float r=30; /*sperm circulation radius*/
float c=0; /*sperm orientation*/
float w=2*M_PI/13; /*sperm rotation per unit time*/
float ed=5000; /*radius of defined area*/
float egg=100; /*egg radius*/
float gc=300000; /*Number of active GC*/
double max=0.0000000001; /*[chemoattractant]s*/
float bind=0.00000000065; /*K1/2*/
float binds=0.00000000008; /*SD of K1/2*/
float hill=0.49; /*Hill coefficient*/
float hills=0.03; /*SD of Hill coefficient*/
float cGMPm=4.3; /*generation of cGMP per active GC per unit time*/
float cGMPd=1.7; /*SD of cGMP generation*/
int thr=100;/*Threshold for initiation of turning by cGMP*/
int d=2000;
float curv[2][15]={{-0.238842,-6.041005,-12.98229,-18.29885,-21.63054,-23.85485,-24.57967,-
12.09104,34.23337,81.7475,103.0932,119.3109,123.068,118.7977,110.3706},
{24.90107,30.74057,33.15464,32.31918,29.85375,25.61568,13.72072,-10.4685,-46.42694,-52.19626,-35.22384,-
12.64072,7.442462,22.09331,31.02494}};
/*describing turning trajectory*/

float x,y; /*central coordinate of sperm cirular motion*/
float a,b; /*sperm coordinate*/
double conc; /*local [chemoattractant]*/
double q; /*used for binding calculation*/
float h,K,H,gcbsum,cGMP ; /*used for binding calculation*/
float gcb[3]={0,0,0}; /*memories for counting of GC binding*/
float cir[2][15]; /*memories for turning*/
int m,reach; /*used for counting*/

/*setting for random digits*/
init_genrand((unsigned)time(NULL));

/*pre-calculation for [chemoattractant] gradient*/
h=max/(log(egg/ed));


/*Here, we can input the initial sperm location and orientation*/
```

```
t=0;
x=500;
y=0;
c=0;

/*calculation for initial sperm coordinate*/
a=x+r*cos(c);
b=y+r*sin(c);

/*calculation for chemoattractant binding*/
conc=h*(log((sqrt(a*a+b*b))/ed));
K=rand_normal(bind,binds);
H=rand_normal(hill,hills);
cGMP=rand_normal(cGMPm,cGMPd);
q=(pow(conc,H))/(pow(K,H)+pow(conc,H));
gcb[0]=q*gc/d;
gcb[1]=0;
gcb[2]=0;

  /*repeated simulation for 30,000 unit time (30 min)*/
  for(t=1;t<30000;t++)
    {

      /*counting chemoattractant binding to GC*/
      gcbsum=0;
      for(m=0;m<2;m++)
        {
          gcbsum+=gcb[m];
        }

      /*Here sperm decides whether turn or not*/
      if(thr<gcbsum*cGMP)
        {
        /*If turn occurs, following transaction is conducted*/

          /*calculation for sperm relocation during the turn behavior*/
          for(m=0;m<15;m++)
            {
              cir[0][m]=cos(c)*curv[0][m]-sin(c)*curv[1][m];
              cir[1][m]=sin(c)*curv[0][m]+cos(c)*curv[1][m];
```

```
    }
for(m=0;m<15;m++,t++)
    {

        /*detection of sperm arrival to the egg*/
        if(rch(a+cir[0][m],b+cir[1][m]) == 1)
          {
              reach=1;
              break;
          }
        c+=w;
        if(c>(2*M_PI))
          {
              c-=(2*M_PI);
          }

          /*GC desensitization*/
          gc-=gcb[2];
          gcb[2]=gcb[1];
          gcb[1]=gcb[0];

          /*calculation for chemoattractant binding*/
          conc=h*(log((sqrt(pow(a+cir[0][m],2)+pow(b+cir[1][m],2)))/ed));
          K=rand_normal(bind,binds);
          H=rand_normal(hill,hills);
          q=(pow(conc,H))/(pow(K,H)+pow(conc,H));
          gcb[0]=(q*gc-gcb[1]-gcb[2])/d;
    }
x=a+cir[0][14]-r*cos(c);
y=b+cir[1][14]-r*sin(c);

/*refractory period (6 unit time (300 msec))*/
for(m=0;m<5;m++,t++)
    {

        /*circular motion*/
        c+=w;
        if(c>(2*M_PI))
          {
              c-=(2*M_PI);
```

```
            }
         a=x+r*cos(c);
         b=y+r*sin(c);

         /*GC desensitization*/
         gc-=gcb[2];
         gcb[2]=gcb[1];
         gcb[1]=gcb[0];

         /*calculation for chemoattractant binding*/
         conc=h*(log((sqrt(a*a+b*b))/ed));
         K=rand_normal(bind,binds);
         H=rand_normal(hill,hills);
         q=(pow(conc,H))/(pow(K,H)+pow(conc,H));
         gcb[0]=(q*gc-gcb[1]-gcb[2])/d;

         /*detection of sperm arrival to the egg*/
         if(rch(a,b) == 1)
            {
               reach=1;
               break;
            }
       }

     t--;
   }

/*If turn does not occur, following transacion is conducted*/
else
   {

     /*circular motion*/
     c+=w;
     if(c>(2*M_PI))
       {
         c-=(2*M_PI);
       }
     a=x+r*cos(c);
     b=y+r*sin(c);
```

```
                /*GC desensitization*/
                gc-=gcb[2];
                gcb[2]=gcb[1];
                gcb[1]=gcb[0];

                /*calculation for chemoattractant binding*/
                conc=h*(log((sqrt(a*a+b*b))/ed));
                K=rand_normal(bind,binds);
                H=rand_normal(hill,hills);
                q=(pow(conc,H))/(pow(K,H)+pow(conc,H));
                gcb[0]=(q*gc-gcb[1]-gcb[2])/d;

                /*detection of sperm arrival to the egg*/
                if(rch(a,b) == 1)
                  {
                    reach=1;
                    break;
                  }

            }
        }
    return 0;
}
```

Supporting Figure 2

Effect of field size

Simulations with several field sizes were performed to elucidate the effects of the field size. As shown in the Figure, simulations in 2500, 5000, and 7500 μm fields showed similar results, suggesting that the field size does not significantly affect the results.

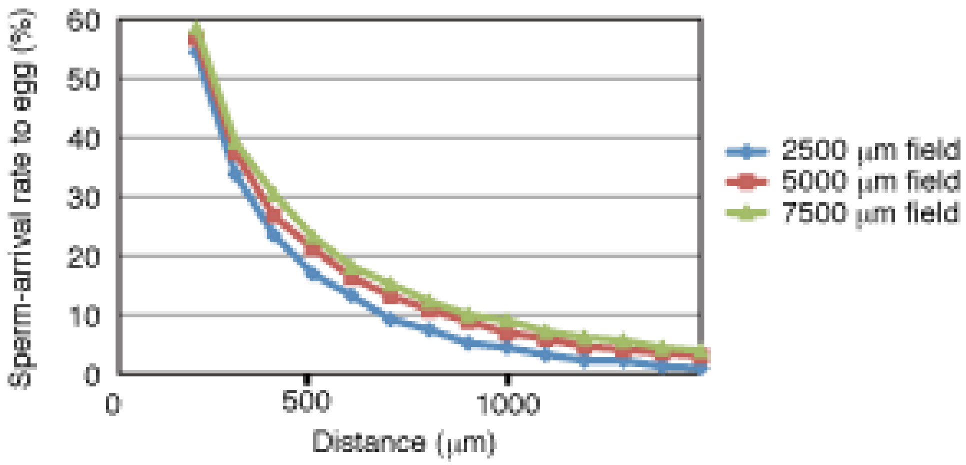

Effects of field size in simulation. Simulation results for 2500, 5000, and 7500 μm fields with the sea urchin model are shown in the graph. The x-axis indicates the distance between the sperm start-point and the center of the egg, and egg-arrival rates of sperm from that point are shown as the longitudinal axis (n = 10000). The length of the simulation was 30 min.

Supporting Information 3

Source code of the chemotaxis simulation with starfish model

```
#include <stdio.h>
#include <math.h>
#include <stdlib.h>
#include <time.h>
#include "MT.h"

/*This function detects the arrival of sperm to the egg*/
double rch(float a, float b)
{
    if(sqrt(a*a+b*b) < 100)
        {
            return 1;
        }
    else
        {
            return 0;
        }
}

/*This function generates random number with normal distribution*/
double rand_normal(double mu, double sigma)
{
    double z;
    double a;
    double b;
    a=genrand_real3();
    b=genrand_real3();
    z=sqrt(-2*log(a))*sin(2*M_PI*b);
    return mu+sigma*z;
}

/*This is a main function for simulation*/
int main(void)
{
```

```
/*declaration of variables*/
    int t=0; /*unit time*/
    float r=41; /*sperm circulation radius*/
    float c=0; /*sperm orientation*/
    float w=2*M_PI/13; /*sperm rotation per unit time*/
    float ed=5000; /*radius of defined area*/
    float egg=100; /*egg radius*/
    float gc=110000; /*Number of active GC*/
    double max=0.0000000001; /*[chemoattractant]s*/
    float bind=0.00000000065; /*K1/2*/
    float binds=0.00000000008; /*SD of K1/2*/
    float hill=0.49; /*Hill coefficient*/
    float hills=0.03; /*SD of Hill coefficient*/
    float cGMPm=4.3; /*generation of cGMP per active GC per unit time*/
    float cGMPd=1.7; /*SD of cGMP generation*/
    int thr=100;/*Threshold for initiation of turning by cGMP*/
    int d=2000;
    float curv[2][15]={{-0.399517,-7.576554,-16.22961,-26.53564,-28.18429,-28.09471,-25.17963,-
4.056847,24.12319,39.15945,46.54345,51.62645,46.84994,33.94788},
{33.6913,40.73724,43.72241,42.69501,39.7695,36.36147,33.36521,27.1607,9.171787,4.704552,17.05667,27.89742,49.50063,67.
45731,82.02077}};
/*describing turning trajectory*/


    float x,y; /*central coordinate of sperm cirular motion*/
    float a,b; /*sperm coordinate*/
    double conc; /*local [chemoattractant]*/
    double q; /*used for binding calculation*/
    float h,K,H,gcbsum,cGMP ; /*used for binding calculation*/
    float gcb[2]={0,0}; /*memories for counting of GC binding*/
    float cir[2][15]; /*memories for turning*/
     int m,reach; /*used for counting*/

    /*setting for random digits*/
    init_genrand((unsigned)time(NULL));

    /*pre-calculation for [chemoattractant] gradient*/
    h=max/(log(egg/ed));



    /*Here, we can input the initial sperm location and orientation*/
```

```
t=0;
x=500;
y=0;
c=0;

/*calculation for initial sperm coordinate*/
a=x+r*cos(c);
b=y+r*sin(c);

/*calculation for chemoattractant binding*/
conc=h*(log((sqrt(a*a+b*b))/ed));
K=rand_normal(bind,binds);
H=rand_normal(hill,hills);
cGMP=rand_normal(cGMPm,cGMPd);
q=(pow(conc,H))/(pow(K,H)+pow(conc,H));
gcb[0]=q*gc/d;
gcb[1]=0;

  /*repeated simulation for 30,000 unit time (30 min)*/
  for(t=1;t<30000;t++)
    {

      /*counting chemoattractant binding to GC*/
      gcbsum=0;
      for(m=0;m<2;m++)
        {
          gcbsum+=gcb[m];
        }

      /*Here sperm decides whether turn or not*/
      if(thr<gcbsum*cGMP)
        {
        /*If turn occurs, following transaction is conducted*/

          /*GC desensitization*/
          gc-=gcbsum;
          gcb[0]=0;
          gcb[1]=0;

          /*calculation for sperm relocation during the turn behavior*/
```

```
for(m=0;m<15;m++)
    {
        cir[0][m]=cos(c)*curv[0][m]-sin(c)*curv[1][m];
        cir[1][m]=sin(c)*curv[0][m]+cos(c)*curv[1][m];
    }
for(m=0;m<15;m++,t++)
    {

        /*detection of sperm arrival to the egg*/
        if(rch(a+cir[0][m],b+cir[1][m]) == 1)
            {
                reach=1;
                break;
            }
        c+=w;
        if(c>(2*M_PI))
            {
                c-=(2*M_PI);
            }

        /*calculation for chemoattractant binding*/
        gcb[1]+=gcb[0];
        conc=h*(log((sqrt(pow(a+cir[0][m],2)+pow(b+cir[1][m],2)))/ed));
        K=rand_normal(bind,binds);
        H=rand_normal(hill,hills);
        q=(pow(conc,H))/(pow(K,H)+pow(conc,H));
        gcb[0]=q*gc/d;
    }
x=a+cir[0][14]-r*cos(c);
y=b+cir[1][14]-r*sin(c);

/*refractory period (6 unit time (300 msec))*/
for(m=0;m<5;m++,t++)
    {

        /*circular motion*/
        c+=w;
        if(c>(2*M_PI))
            {
                c-=(2*M_PI);
```

```
            }
          a=x+r*cos(c);
          b=y+r*sin(c);

          /*calculation for chemoattractant binding*/
          gcb[1]+=gcb[0];
          conc=h*(log((sqrt(a*a+b*b))/ed));
          K=rand_normal(bind,binds);
          H=rand_normal(hill,hills);
          q=pow(conc,H)/(K+pow(conc,H));
          gcb[0]=q*gc/d;

          /*detection of sperm arrival to the egg*/
          if(rch(a,b) == 1)
            {
              reach=1;
              break;
            }
        }

      t--;
    }

/*If turn does not occur, following transacion is conducted*/
else
    {

      /*circular motion*/
      c+=w;
      if(c>(2*M_PI))
        {
          c-=(2*M_PI);
        }

      /*calculation for chemoattractant binding*/
      a=x+r*cos(c);
      b=y+r*sin(c);
      gcb[1]+=gcb[0];
      conc=h*(log((sqrt(a*a+b*b))/ed));
      K=rand_normal(bind,binds);
```

```
            H=rand_normal(hill,hills);
            q=(pow(conc,H))/(pow(K,H)+pow(conc,H));
            gcb[0]=q*gc/d;

            /*detection of sperm arrival to the egg*/
            if(rch(a,b) == 1)
              {
                reach=1;
                break;
              }

          }
      }
    return 0;
}
```

Supporting Information 4

Effect of radius

To clarify whether the difference in swimming radius generates differences between the performance of sea urchin and starfish models, we conducted simulations with a hybrid sperm model, which was basically the same as the sea urchin model, except for having the swimming radius of starfish. As shown in the Figure, the sea urchin model with starfish radius (red) showed similar results to the original sea urchin model (blue), but not the starfish model (green), although the egg-arrival ratio of the hybrid model from the 500 μm distance was remarkably higher than that of the other models. Therefore, the swimming radius affected only the results of the simulations, in which the sperm start position was close to the egg.

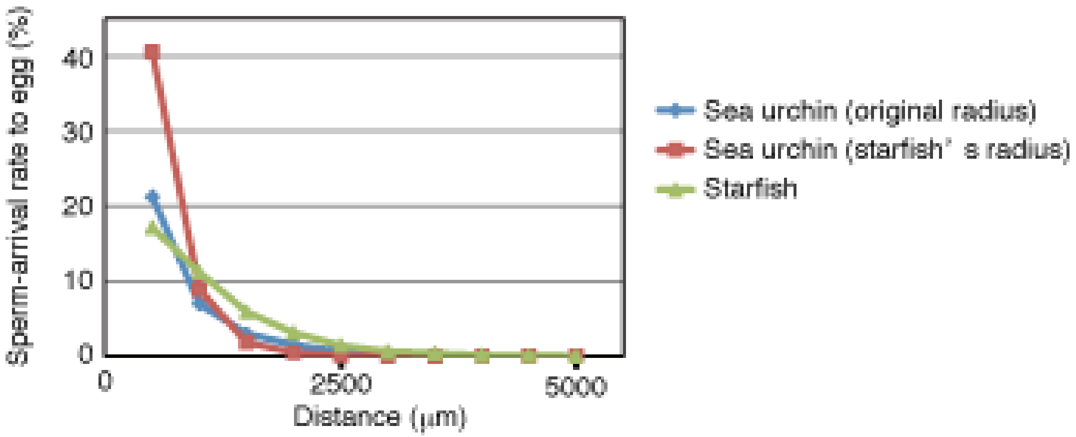

Regarding the effects of radius in the simulation, the red marker and line represent the simulation results of the sea urchin model with the radius of the starfish, where the x- and y-axes are the same as in Fig. 3A. Blue and green symbols represent the results of the sea urchin and starfish model with original parameters, respectively.

Supporting Information 5

Analysis of the pathway for GC dephosphorylation

For analysis of the GC dephosphorylation pathway, we used mobility shifts of GC in electrophoresis, which is associated with a loss of phosphate groups by Asap treatment. Western blotting using an anti-GC antibody has been used to visualize the shift, and it is known that okadaic acid (OA), a PP2A inhibitor, prevents dephosphorylation and ceases this shift caused by Asap [25]. Sperm samples exposed to Asap in the presence of the PKC inhibitor GF109203X (GFX) showed significant inhibition of GC dephosphorylation by Asap, similar to that with OA (A). This result indicated that PKC and PP2A activities are essential for GC dephosphorylation, although it is unclear whether PKC is directly related to PP2A. In contrast, the PKC activator phorbol 12-myristate 13-acetate (PMA) could not independently induce GC dephosphorylation by Asap (B). Furthermore, IBMX, which inhibits phosphodiesterase (PDE) activity, and consequently, activates downstream signaling from the CNGK channel, was unable to evoke dephosphorylation (B), suggesting that GC dephosphorylation requires Asap binding to GC. However, Asap did not induce GC dephosphorylation in a 10-fold concentration of K^+ artificial seawater (ASW), where K^+ efflux through CNGK was inhibited by external K^+, indicating that Asap binding alone was still not sufficient to induce GC dephosphorylation. The combination of PMA and Asap could induce GC dephosphorylation in high K^+ ASW (C), suggesting that GC dephosphorylation required both Asap binding and PKC activation. These results support the assumption that the GC of starfish has characteristics similar to human NPR.

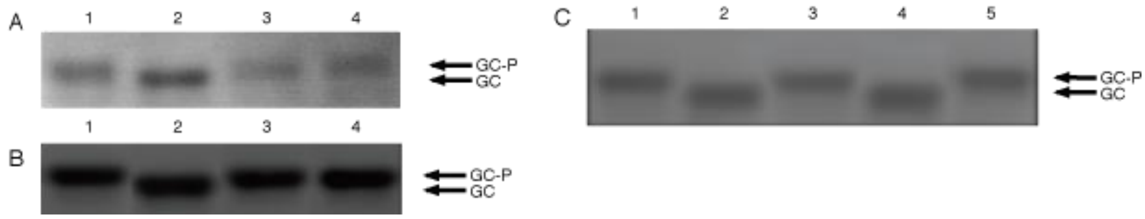

A–C: GCs of starfish sperm visualized by western blotting using an anti-GC antibody showing the phosphorylation status. "GC-P" and "GC" indicate GC with and without phosphorylation, respectively. Lanes 1 and 2 (A-C): negative control (non-treated sperm) and positive control (Asap-treated sperm), respectively. A: PKC-PP2A pathway inhibition. Lanes 3 and 4 show the results for sperm treated with Asap with GFX and OA, respectively. B: PKC and CNGK activation. Lanes 3 and 4 represent the results for sperm treated with PMA and IBMX, respectively. C: The experiment with high K^+ ASW. Lane 3 is sperm treated with Asap in 10-fold-high K^+ ASW. Lanes 4 and 5 show "PMA and Asap-treated" sperm and PMA-treated sperm, respectively, in high K^+ ASW.

Material and Method

A. amurensis was collected from Otsuchi Bay and Tokyo Bay in Japan, and Sandy Bay in Tasmania, Australia. Dry sperm were obtained from the testes and kept on ice.

10% SDS-PAGE was conducted, followed by western blotting using an anti-GC antibody. The molecular weight of GC was approximately 120 kDa, and both phosphorylated and dephosphorylated GC appeared to be approximately of the same size. Sample preparation and detection were performed as previously described (Kawase et al., 2004). Asap, IBMX, PMA, and GFX were used at a final concentration of 1 M in ASW and 5 μM OA in ASW containing 0.01% digitonin.

Supporting Information 6

Analysis of Long-range Chemotaxis by the Fertilization assay

Since chemotaxis is an essential phenomenon for successful fertilization, if the desensitization contributes to the chemotaxis, a proper desensitization would be required for fertilization. Therefore, a “Fertilization assay” was developed to examine the role of GC dephosphorylation in the fertilization. For the Fertilization assay, the mass of sperm was added at a distance of 1 or 5 cm away from the eggs, and egg fertilization rates were counted 30 min later. From 1 cm, every sperm group pre-treated with Asap, IBMX, PMA, GFX, or OA was able to fertilize eggs with high efficiency as well as the control, indicating that the sperm fertility was not disturbed by any of those inhibitors. However, sperm groups pre-treated with each of these chemicals showed obvious decreases in the fertilization rates from 5 cm distance, although only 20% decrease was observed in the control. In particular, treatment by PMA, GFX, or OA resulted in extensive reduction of the fertilization rate, whereas sperm groups treated by Asap or IBMX still retained about 20% fertilization rates, suggesting that the properly controlled GC dephosphorylation, namely sperm desensitization, is required for the successful chemotaxis although these results can’t deny the possibility that chemicals affect to the sperm mobility itself.

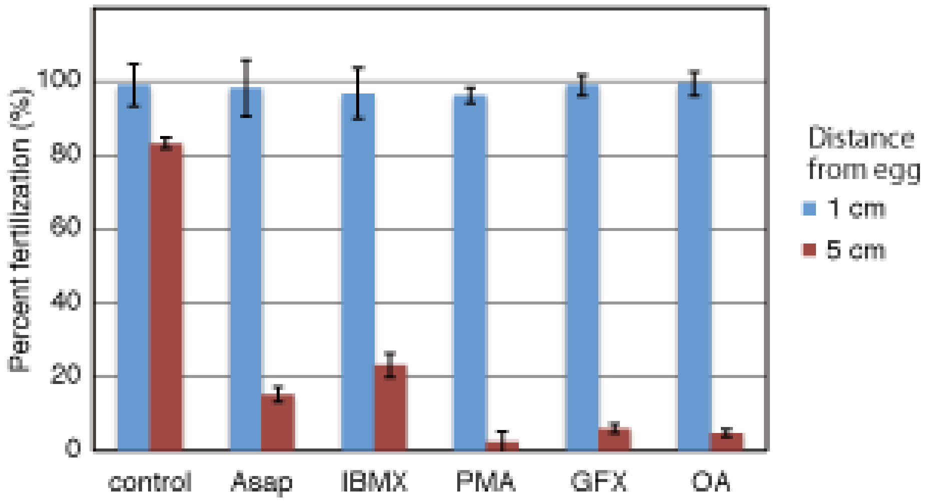

The y-axis indicates fertilization rate of the eggs in each condition. The data are expressed as the mean ± SD (n = 6). Control means intact sperm and other samples were treated with the corresponding reagents: Asap, IBMX, PMA, GFX, and OA. In all cases, sperm were able to fertilize the egg from a distance of 1 cm (blue bars), although only control sperm could well-fertilize the egg from a distance of 5 cm (red bars).

Material and Method

Mature eggs were prepared by treating the ovaries with 10 µM 1-methyladenine. Dry sperm, obtained same as SI 5, was diluted with ASW, containing each of the chemical reagents, to approximately 10^6 cells/µL in 500 µL, and incubated 15 min on ice. Concentrations of each chemical reagent were same as dephosphorylation analysis in SI 5. Subsequently, the suspensions were centrifuged at 1000 × *g* and the pellets were re-suspended in a small volume of ASW as sperm samples. Approximately 40 matured eggs were prepared for each sperm sample and carefully layed down the centers of 20-cm glass dishes filled with seawater. Sperm samples were then gently added either 1 cm or 5 cm away from the eggs. After 30 min, fertilization rates of the eggs were counted.
